# Influence of eye biometrics and corneal micro-structure on noncontact tonometry

**DOI:** 10.1371/journal.pone.0177180

**Published:** 2017-05-04

**Authors:** Danilo A. Jesus, Małgorzata Majewska, Patrycja Krzyżanowska-Berkowska, D. Robert Iskander

**Affiliations:** 1 Department of Biomedical Engineering, Wroclaw University of Science & Technology, Wroclaw, Poland; 2 Department of Ophthalmology, Wroclaw Medical University, Wroclaw, Poland; Bascom Palmer Eye Institute, UNITED STATES

## Abstract

**Purpose:**

Tonometry is widely used as the main screening tool supporting glaucoma diagnosis. Still, its accuracy could be improved if full knowledge about the variation of the corneal biomechanical properties was available. In this study, Optical Coherence Tomography (OCT) speckle statistics are used to infer the organisation of the corneal micro-structure and hence, to analyse its influence on intraocular pressure (IOP) measurements.

**Methods:**

Fifty-six subjects were recruited for this prospective study. Macro and micro-structural corneal parameters as well as subject age were considered. Macro-structural analysis included the parameters that are associated with the ocular anatomy, such as central corneal thickness (CCT), corneal radius, axial length, anterior chamber depth and white-to-white corneal diameter. Micro-structural parameters which included OCT speckle statistics were related to the internal organisation of the corneal tissue and its physiological changes during lifetime. The corneal speckle obtained from OCT was modelled with the Generalised Gamma (GG) distribution that is characterised with a scale parameter and two shape parameters.

**Results:**

In macro-structure analysis, only CCT showed a statistically significant correlation with IOP (R^2^ = 0.25, *p*<0.001). The scale parameter and the ratio of the shape parameters of GG distribution showed statistically significant correlation with IOP (R^2^ = 0.19, *p*<0.001 and R^2^ = 0.17, *p*<0.001, respectively). For the studied group, a weak, although significant correlation was found between age and IOP (R^2^ = 0.053, *p* = 0.04). Forward stepwise regression showed that CCT and the scale parameter of the Generalised Gamma distribution can be combined in a regression model (R^2^ = 0.39, *p*<0.001) to study the role of the corneal structure on IOP.

**Conclusions:**

We show, for the first time, that corneal micro-structure influences the IOP measurements obtained from noncontact tonometry. OCT speckle statistics can be employed to learn about the corneal micro-structure and hence, to further calibrate the IOP measurements.

## Introduction

Glaucoma is the leading cause of global irreversible blindness [1]. It affects more than 70 million people worldwide and it is estimated to reach 111.8 million in 2040 [2]. Although the pathogenesis of glaucoma is not fully understood, the increased level of intraocular pressure (IOP) has been clearly linked to a progressive degeneration of retinal ganglion cells [3,4].

Intraocular pressure is routinely assessed by applanation tonometry and its normal level ranges from about 10 to 21 *mmHg* [5]. The accuracy of applanation tonometry is influenced by corneal stiffness that varies with a number of parameters, such as thickness, curvature and age [6]. The influence of the central corneal thickness (CCT) on IOP measurements has been assessed in several studies in a relationship that has not been precisely specified. A number of corrections has been presented in the literature ranging from 0.12 *mmHg*/0.01*mm* to 1 *mmHg*/0.01*mm* of corneal thickness [7–9]. It is well known that for healthy eyes non-corrected IOP is underestimated in thinner corneas and overestimated in thicker ones. The relationship between anterior corneal radius (CR) and IOP has also been studied [10,11]. Unlike CCT, the effect of CR is small and limited to a range between 0.57 mmHg/1*mm* to 1.14 mmHg/1*mm* [6]. Both CCT and CR may be viewed as corneal macro-structural parameters that influence the accuracy of applanation tonometry. However, *in vitro* experiments [12,13] and theoretical models [14] have shown that the cornea exhibits mechanical and viscoelastic properties. This may explain the wide range obtained for CCT correction and indicate that the macro-structure described by CCT and CR only partially explains the variance of IOP whereas other properties, related to the micro-structure of the cornea, may also contribute [15].

Cornea is found to increase its stiffness as the characteristics of viscoelastic behaviour decrease with age [16,17]. Such changes in corneal micro-structure contribute to a variation of corneal biomechanical variables which may be independent of CCT or IOP. However, the assessment of the micro-structure effect in corneal tissue stiffness was limited to few studies [10] due to the lack of means to measure it *in vivo*. Spoerl *et*. *al* [7] suggested that the measured IOP should be corrected by the CCT and an age-dependent correction factor, pointing that an increase of corneal thickness in a young person has a lower influence on the measured IOP than the same increase of thickness in an older subject.

Despite the assumption made by Spoerl *et*. *al* [7], ageing is not necessarily a linear process and may differently affect each subject. Parameters such as medical history (i.e., diabetes or topical drugs), ocular anatomy, sun exposure, alimentation or ethnicity may all play a role in that process [18,19]. Moreover, the actual correction does not apply to other factors such corneal swelling, wound healing, and diseases such as keratoconus that all have possible effect on corneal micro-structure and hence, subsequently, on the IOP measurements. Therefore, new techniques are needed to measure *in vivo* the contribution of the corneal micro-structure on the IOP measurement.

Optical Coherence Tomography (OCT) has become a popular instrument to visualise the anterior eye. It is based on interferometry which gives rise to speckle—a form of noise originating from the backscattered light that degrades the quality of OCT image. However, speckle also has a signal-carrying component [20] which can be used to infer the micro-structure of the corneal tissue. The texture analysis of the OCT speckle to characterize biological tissues *in vivo* has already been applied in different contexts such as, to differentiate between normal and tumour tissues [21], in diabetic retinopathy characterization [22] and retinal segmentation [23]. Our previous studies have shown that corneal speckle acquired by OCT is also sensitive to micro-structural changes in cornea [24].

Therefore, in this study, the hypothesis was that IOP measurements obtained from noncontact tonometry are influenced by the micro-structure of the cornea. The contribution of the corneal structure on IOP measurements before and after its correction using CCT was explored. Corneal speckle statistics obtained from OCT were used to infer the corneal micro-structure whereas, the macro-structure was analysed using CCT and CR. Subject age was also considered. In addition, other biometric parameters including axial length (AL), anterior chamber depth (ACD) and white-to-white (WTW) corneal diameter were considered for the macro-structure analysis.

## Methods

This is a prospective study that included 56 Caucasian subjects (25 male and 31 female) with a refractive error smaller than 1 Dioptre. A medical history review has been conducted prior to measurements. Only subjects without any reported eye disease were considered. All subjects underwent thorough OCT examination, along standard examination with a biomicroscope, to ensure that their corneas were normal. The number of subjects was sufficient to detect changes of, at least, 6.6% in the mean value of any parameter with power of 1 − *β* = 99% at *α* = 5% level of significance according to the following sample size calculation formula [25]:

$$n=\sigma^{2}\frac{{\left(t\left(1-\alpha ,\;N-2\right)+t\left(1-\beta ,N-2\right)\right)}^{2}}{{\left(\Delta \mu \right)}^{2}}$$

The mean (± standard deviation) age of the participants was 44.7 (± 19.3) years; range from 22 to 78. The study design was approved by the Bioethical Committee of the Wroclaw Medical University (No 332/215). All subjects were treated in accordance with the principles of the Declaration of Helsinki. All subjects provided written informed consent after the goals of the research and consequences of participation had been discussed. Matlab (Mathworks, Natick, MA) was used to develop the algorithm and the statistical analysis, which included the non-parametric Wilcoxon test, the two-sample Kolmogorov-Smirnov test, the coefficient of determination, the linear regression and the multilinear forward step-wise regression analysis based on linear least squares. In this study, only the right eye of each subject was assessed and measured.

### Data acquisition

**Fig 1:**
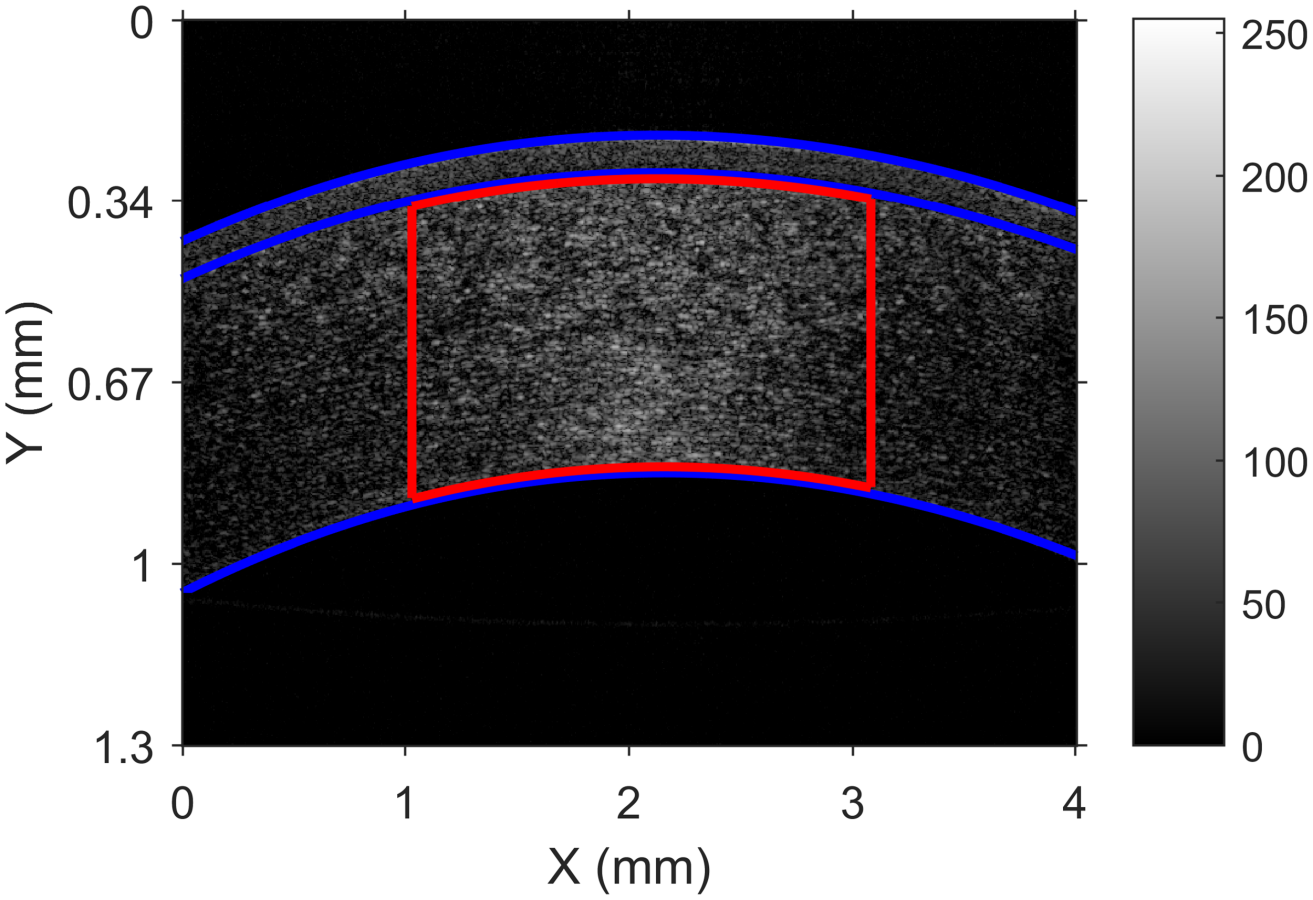
An example of a cropped corneal OCT image and the ROI demarked by the red lines. Blue lines indicate from the top the epithelium, Bowman’s layer and endothelium.

A spectral domain OCT (SOCT, Copernicus HR, Optopol, Poland) was used to acquire images of the cornea. The signal source was a super luminescent diode with a centre wavelength of 850 *nm*, and axial and lateral resolution of 3 *μm* and 15 *μm*, respectively. Each B-scan was sampled through 1024 A-scans with 848 pixels per A-scan. In each image, a region of interest (ROI) consisting of 250 rows and 450 columns was selected for the purpose of studying the corneal speckle, as shown in Fig 1. A simple automatic routine was used to ensure that all ROIs remained at the same position. First, the epithelium, Bowman’s layer and endothelium using a procedure based on image segmentation and edge detection were identified. Then, a ROI was selected according to the established dimensions, considering the null differentiation at epithelium curvature as a reference to the central horizontal position. For the purpose of consistency between images, each cross section was acquired as close as possible to the corneal apex but not at its centre in order to avoid the specular reflection.

Intraocular pressure was measured using the noncontact tonometer Corvis Scheimpflug Technology (Corvis ST, OCULUS, Wetzlar, Germany). In brief, the device applies an air impulse to the corneal apex with a semi-Gaussian distributed intensity during 25 milliseconds. The air puff causes the first applanation point when cornea moves inwards. The cornea gradually returns to its normal convex curvature when the pressure of the air puff decreases, passing through a second applanation point. The IOP evaluation is based on the first applanation time, and it is currently corrected using the pachymetry measurements based on the sectional corneal images taken with an ultra–high-speed Scheimpflug camera and the subject’s age. The Dresden [8] and the Spoerl [7] tables have been suggested as the means to correct the IOP readings in Corvis tonometer. The purpose of those corrections is to reduce the influence of central corneal thickness and aging on IOP readings.

Further, IOLMaster 700 (Carl Zeiss Meditec AG, Jena, Germany) was used to measure CCT, CR, AL, ACD and WTW corneal diameter. All measurements were acquired in a sequence which started with the noncontact tonometer, followed by the IOL Master and finished with the OCT. A break of ten minutes was set between tonometry measurements and the following biometry and OCT acquisitions. To date, IOLMaster 500 has been the gold standard in optical biometry. However, the agreement between the gold standard and the IOLMaster 700 has shown to be excellent [26]. All measurements followed the same acquisition sequence and were acquired at the same time of the day. This was important to maintain the consistency of the results since IOP is dynamic with regular circadian variations and random short-term and long-term fluctuations.

### Data processing

Speckle in OCT images results from the interference of the photons with the tissue. This interaction changes accordingly the relationship between the light source properties and the dimension of the scatters in a sample. Thus, the analysis of speckle statistics can be employed for assessment of the tissue optical properties [27]. This is performed by plotting the pixel intensities within the selected ROI of OCT B-mode scans using linear (not log-compressed) signals and modelling the resultant intensity histogram with an appropriate probability density function. We have previously shown that the corneal signal intensity distributions are optimally modelled by the Generalised Gamma distribution [24]. The Generalised Gamma is a three parameter distribution which includes many well-known distributions as special cases [28]. Its probability density function may be written as:

$$f_{GG}\left(x;a,v,p\right)=\;\left|p\right|x^{pv-1}\cdot \text{exp}\left\{{\left(\frac{x}{a}\right)}^{p}\right\}/a^{pv}\Gamma \left(v\right)$$

**Fig 2:**
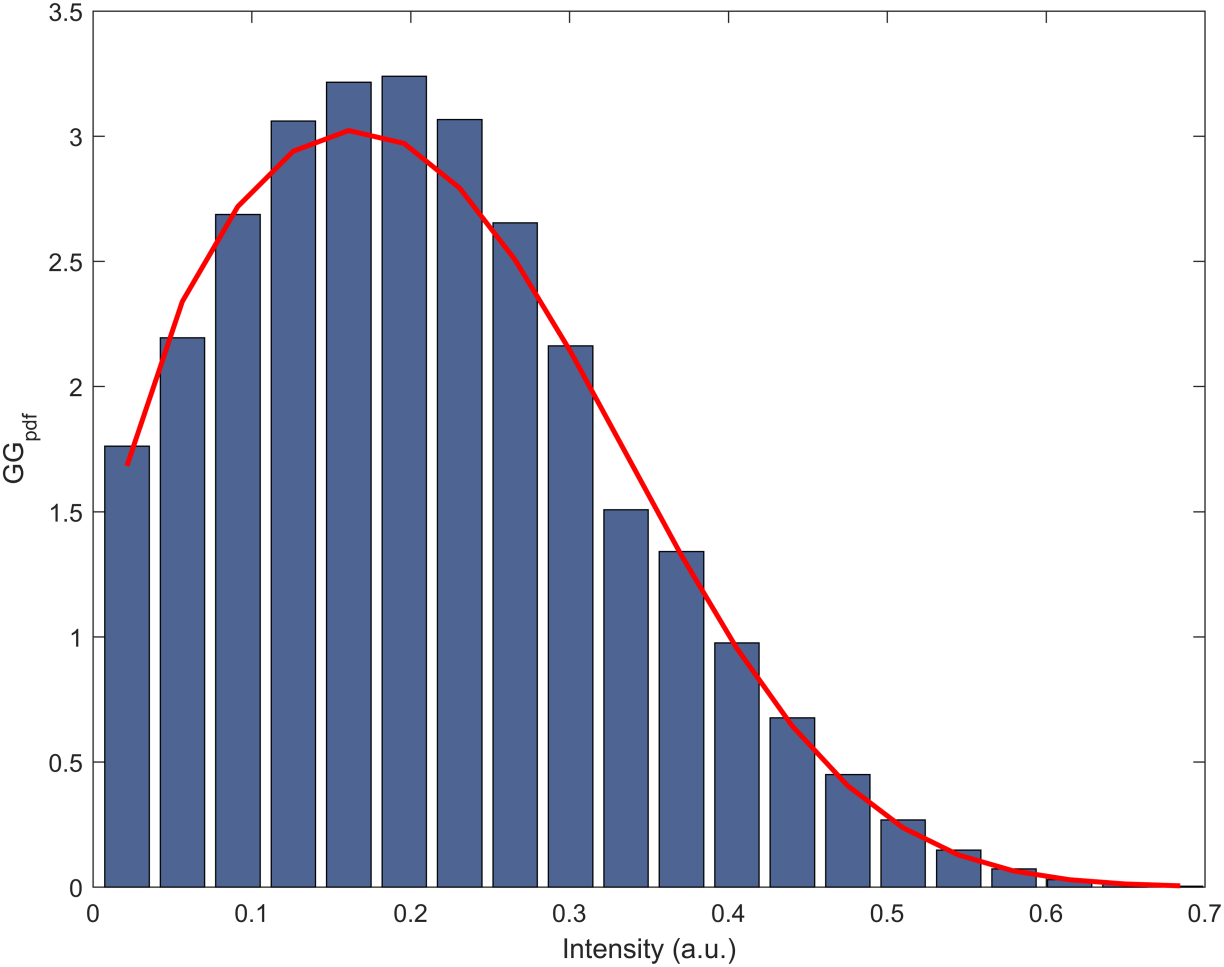
An example of a corneal intensity histogram with 20 bins and the superimposed Generalised Gamma probability density function.

Where *a* is the scale parameter, *v* and *p* are the shape parameters, *x* is the pixel intensity and *Γ*(.) represents the conventional Gamma function [29]. The parameters *a*, *v* and *p* were estimated using the method of maximum likelihood [30]. Fig 2 shows an example of an intensity histogram from a corneal ROI modelled with the Generalised Gamma function.

## Results

The analyses of the influence of the structural parameters on the IOP measurements are presented in two sections, focusing on macro and micro-structure. Macro-structural parameters include CCT, CR, AL, ACD and WTW corneal diameter, whereas the micro-structural parameters are represented by the corneal speckle statistics.

### Macro-structure

**Fig 3:**
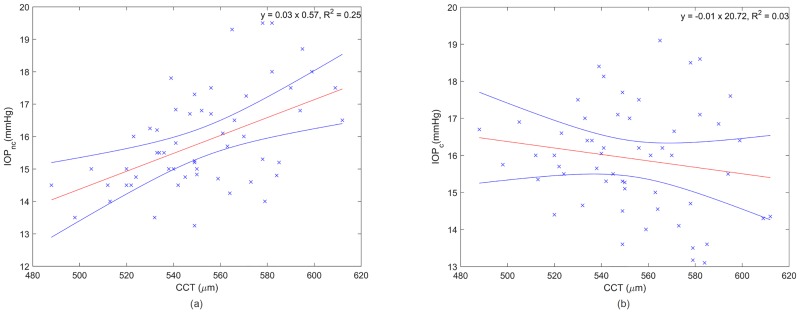
Non-corrected (a) and corrected (b) IOP measurements as a function of CCT acquired with IOLMaster where the red and blue lines are the linear regression and the confidence interval at 95%, respectively.

Fig 3 shows the influence of CCT on non-corrected (a) and corrected (b) IOP values. Both IOP values are provided by the tonometer Corvis ST. A significant correlation (R^2^ = 0.25, p<0.001) is observed in Fig 3a whereas in 3b no correlation is observed (R^2^ = 0.028, p = 0.11). The results are in agreement with the expectations since Corvis ST uses CCT as a correction factor based on the Dresden table [8].

**Fig 4:**
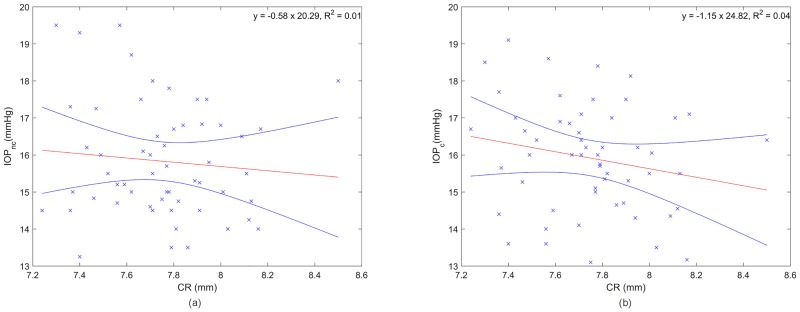
Non-corrected (a) and corrected (b) IOP measurements as a function of CR where the red and blue lines are the linear regression and the confidence interval at 95%, respectively.

Fig 4 shows the association between CR and IOP for non-corrected (a) and corrected (b) values. In none of the cases a significant correlation was observed (p>0.05).

Table 1 presents a summary of the regression analysis for the macro-structural parameters presented above. In addition, correlation between IOP measurements and other parameters acquired with the IOL Master are presented. None of them, except of CCT for the non-corrected IOP, has shown to influence the IOP measurements (*p*>0.05) for the sampled group.

**Table 1:** Macro-structural parameters and their correlations with non-corrected and corrected IOP values.

Parameter (units)	Minimum	Maximum	Mean ± SD	Non-corrected IOP		Corrected IOP	
				R^2^	*p*-value	R^2^	*p*-value
CCT (μm)	488.00	612.00	552.75 ± 27.89	0.250	**<0.001**	0.028	0.107
CR (mm)	7.24	8.50	7.75 ± 0.25	0.009	0.241	0.042	0.065
AL (mm)	21.64	24.88	23.29 ± 0.82	0.037	0.076	0.002	0.372
ACD (mm)	2.58	4.14	3.28 ± 0.38	0.025	0.122	0.029	0.104
WTW (mm)	11.40	12.70	11.99 ± 0.32	0.047	0.053	0.012	0.205

### Micro-structure and age

**Fig 5:**
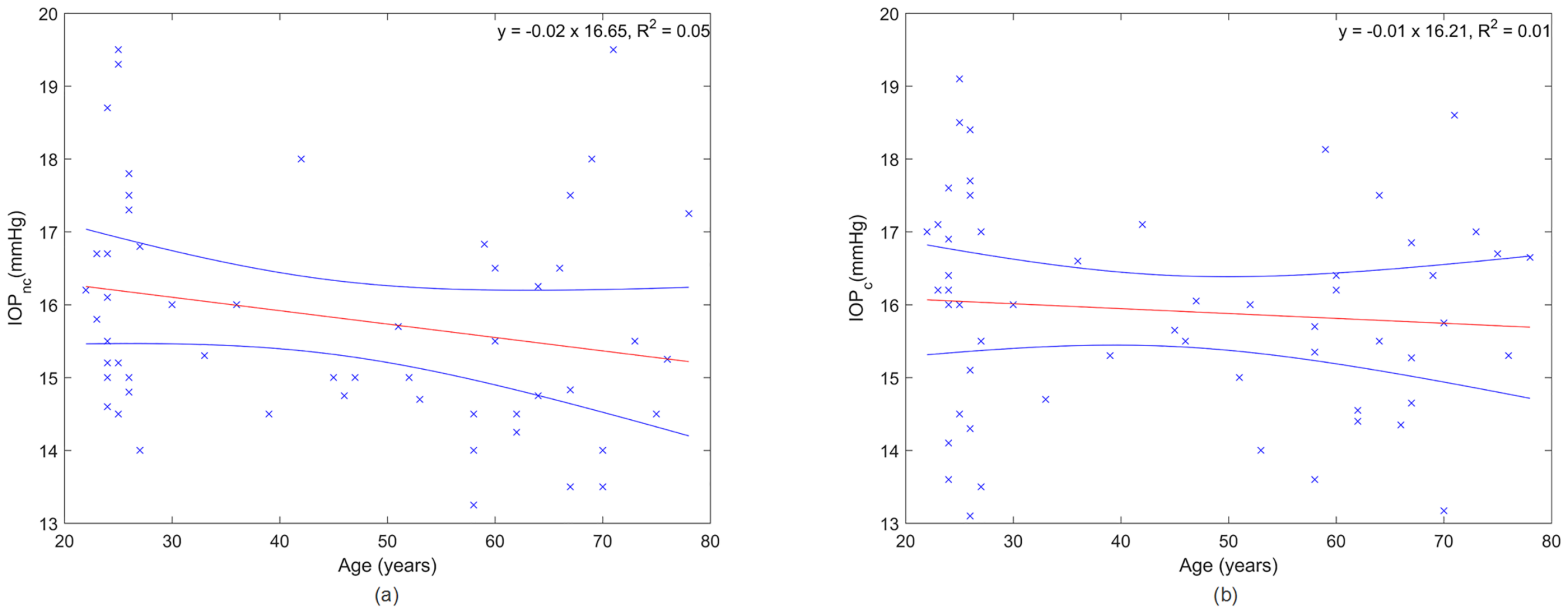
Age influence on non-corrected (a) and corrected (b) IOP measurements where the red and blue lines are the linear regression and the confidence interval at 95%, respectively.

Age has recently been proposed as parameter to access the corneal micro-structural properties and hence, to correct the IOP values [31]. Fig 5a shows that age presents a weak correlation even though significant for uncorrected IOP (R^2^ = 0.05, *p* = 0.04). However, this significant correlation is no longer valid for corrected IOP with CCT (R^2^ = 0.008, *p* = 0.25), as shown in Fig 5b.

**Fig 6:**
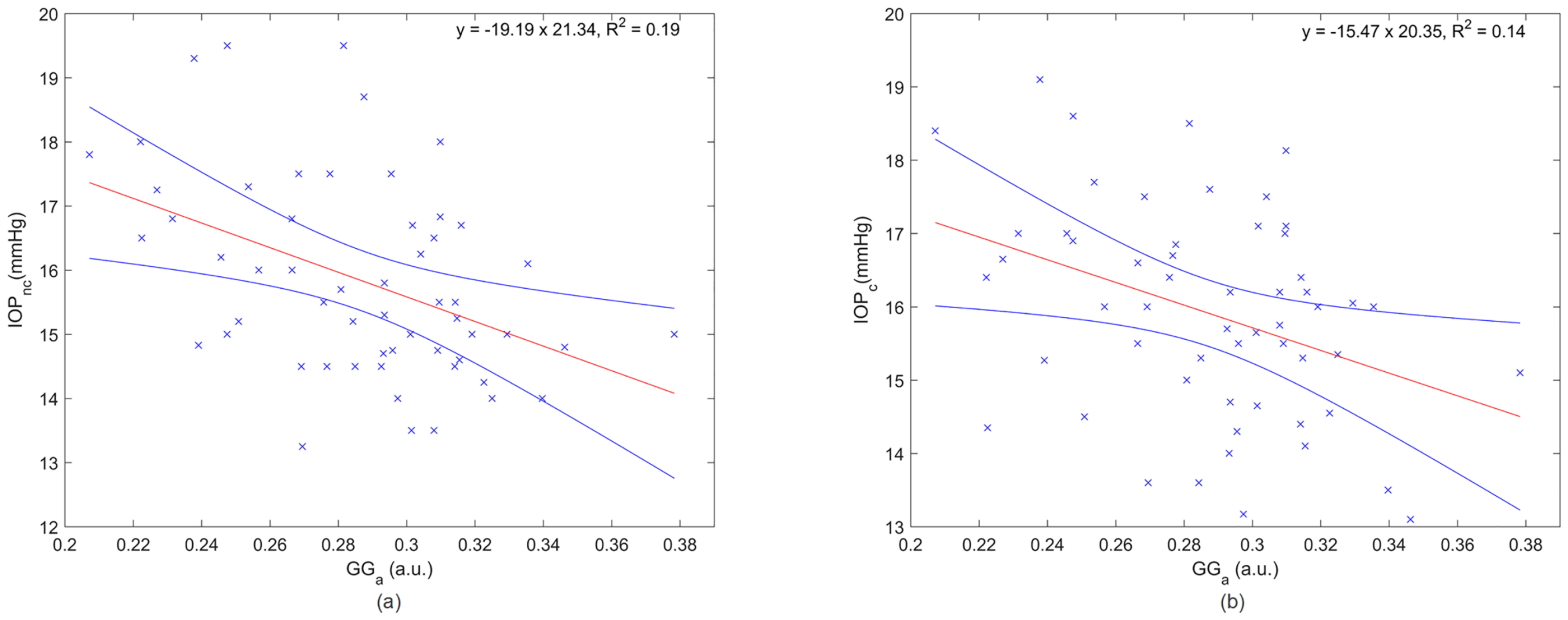
Non-corrected (a) and corrected (b) IOP as a function of the scale parameter *(a)* of the Generalised Gamma distribution where the red and blue lines are the linear regression and the confidence interval at 95%, respectively.

Fig 6 shows the influence of the corneal micro-structure on IOP measurements considering the speckle analysis. Non-corrected (a) and corrected (b) IOP are plotted as a function of the scale parameter of the Generalised Gamma distribution which has shown to be the most sensitive parameter to the IOP variation (see Table 2). Similarly to CCT, a statistically significant correlation before the correction (R^2^ = 0.19, *p*<0.001) was observed. The correlation remained significant (R^2^ = 0.14, *p* = 0.002) for the corrected IOP.

**Table 2:** Micro-structure parameters and their influence on non-corrected and corrected IOP values.

Parameter (units)	Minimum	Maximum	Mean ± SD	Non-corrected IOP		Corrected IOP	
				R^2^	*p*-value	R^2^	*p*-value
Age (years)	22	78	44.66 ± 19.32	0.053	**0.043**	0.008	0.255
*GG*_*a*_ (a.u.)	0.21	0.38	0.287 ± 0.035	0.190	**<0.001**	0.141	**0.002**
*GG*_*v*_ (a.u.)	0.37	0.52	0.437 ± 0.029	0.133	**0.002**	0.074	**0.021**
*GG*_*p*_ (a.u.)	2.28	3.41	2.80 ± 0.27	0.183	**<0.001**	0.069	**0.025**
*GG*_*v/p*_ (a.u.)	0.11	0.23	0.158 ± 0.025	0.166	**<0.001**	0.068	**0.026**

**Fig 7:**
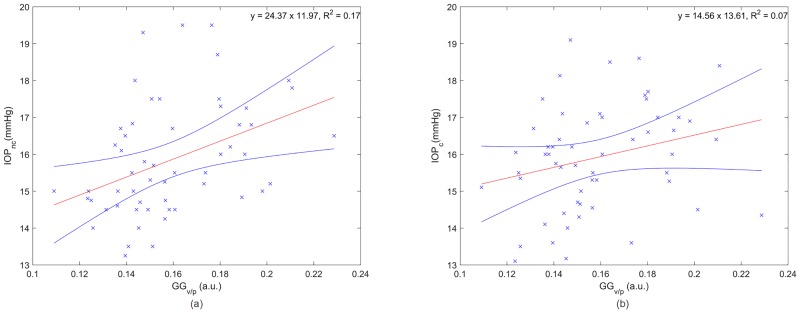
Non-corrected (a) and corrected (b) IOP as a function of the scatter density (*v/p*) based on the Generalised Gamma distribution where the red and blue lines are the linear regression and the confidence interval at 95%, respectively.

The scatter density has been associated to the ratio of the shape parameters (*v/p*) [32]. Fig 7 shows the IOP as a function of the scatter density. A significant relationship was observed for the non-corrected (R^2^ = 0.17, *p*<0.001) and corrected IOP measurements (R^2^ = 0.07, *p* = 0.026).

The influence of micro-structural parameters is summarised in Table 2. The regression analysis of age and each parameter of the Generalised Gamma distribution for non-corrected and corrected IOP are presented.

**Fig 8:**
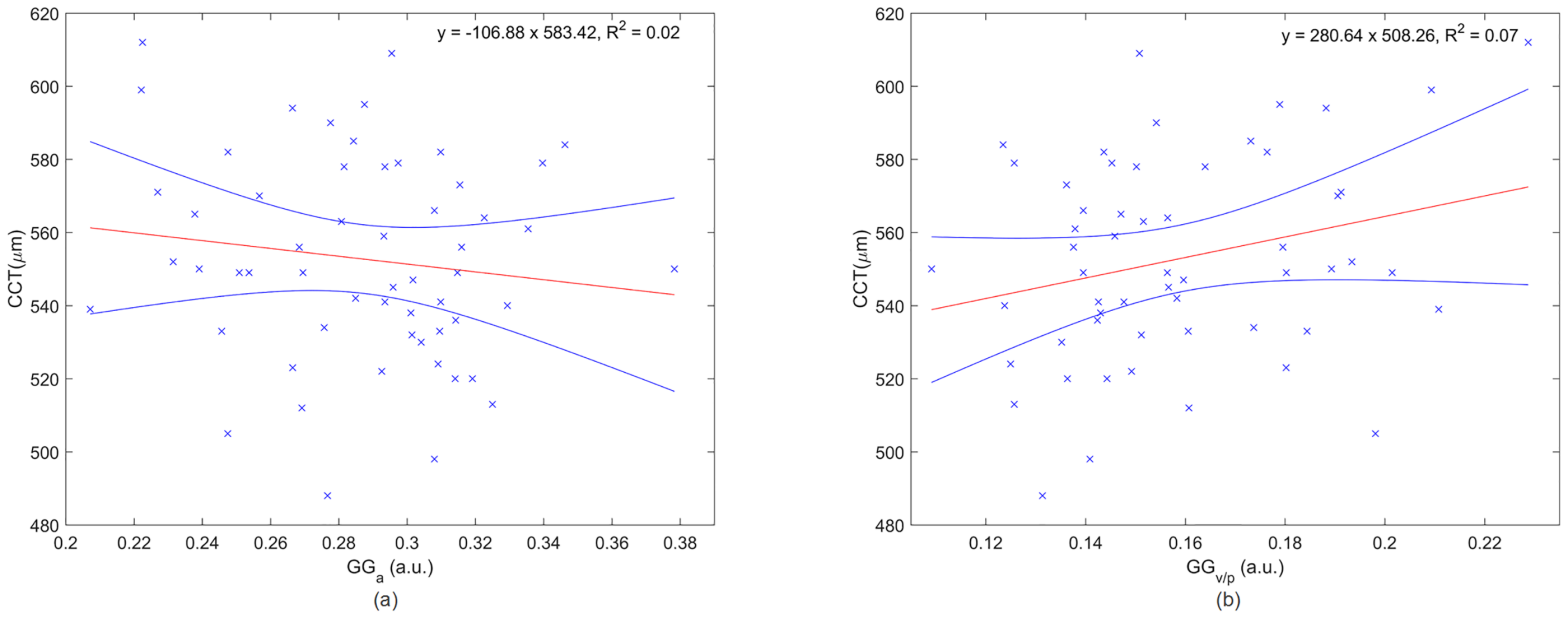
Central corneal thickness as a function of the scale (a) and the coefficient of the shape parameters (b) where the red and blue lines are the linear regression and the confidence interval at 95%, respectively.

Fig 8 shows the relationship between the corneal speckle and CCT. No significant correlation was observed for the Generalised Gamma scale (R^2^ = 0.018, p = 0.16) and the CCT pointing to the fact that backscatter cross section does not depend on CCT. However, the ratio of the shape parameters presented a weak but significant correlation (R^2^ = 0.067, p = 0.027).

**Fig 9:**
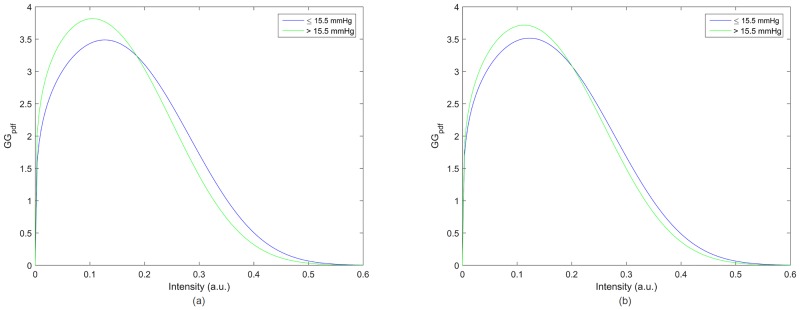
Probability density function of the Generalised Gamma distribution considering two averaged IOP groups for non-corrected (a) and corrected (b) IOP measurements.

The IOP values were averaged into two groups. The first group included all values bellow the IOP median of all subjects, 15.5 *mmHg* (IOP_nc_: 14.66 ± 0.60 *mmHg*; IOP_c_: 14.54 ± 0.77 *mmHg*) whereas the second group took into account the values above the median (IOP_nc_: 17.18 ± 1.12 *mmHg*; IOP_c_: 16.88 ± 0.91 *mmHg*). Fig 9 shows the estimates of the Generalised Gamma probability density functions for the mean values obtained in each group for the non-corrected (a) and corrected (b) IOP. A statistically significant difference between functions was observed for the non-corrected (*p* = 0.007) and corrected IOP values (p = 0.047) accordingly the two sample Kolmogorov-Smirnov test.

The comparison between the two non-corrected IOP groups for each analysed parameter is presented in Table 3. Only CCT and the Generalised Gamma parameters presented a statistically significant difference (*p*<0.05) considering the Wilcoxon test.

**Table 3:** Comparison between the IOP groups corresponding to the values bellow (Group 1) and above (Group 2) the IOP median for each analysed parameter and the respective *p*-value.

Parameter (units)	Group 1 Mean ± SD	Group 2 Mean ± SD	*p*-value
CCT (μm)	560.96 ± 26.70	547.03 ± 27.66	**0.002**
CR (mm)	7.81 ± 0.24	7.71 ± 0.27	0.425
AL (mm)	23.39 ± 0.91	23.23 ± 0.75	0.588
ACD (mm)	3.24 ± 0.37	3.32 ± 0.39	0.863
WTW (mm)	12.07 ± 0.34	11.93 ± 0.30	0.199
Age (years)	46.83 ± 18.45	43.15 ± 20.06	0.119
*GG*_*a*_ (a.u.)	0.30 ± 0.03	0.28 ± 0.03	**0.003**
*GG*_*v*_ (a.u.)	0.43 ± 0.03	0.44 ± 0.02	**0.023**
*GG*_*p*_ (a.u.)	2.85 ± 0.28	2.77 ± 0.27	**0.005**
*GG*_*v/p*_ (a.u.)	0.15 ± 0.03	0.16 ± 0.02	**0.006**

Lastly, forward stepwise regression based on least squares was applied to predict the non-corrected IOP using all macro and micro-structural parameters. It starts with no variables in the model, testing the addition of each one and adding the variable that improves the model the most. The process is repeated until the model cannot be further improved. Among all parameters, only CCT and the scale parameter of the GG were considered significant to the data modelling (R^2^ = 0.39, p<0.001) according to the following regression,

$$IOPnc=0.0248\cdot CCT-16.534\cdot GG_{a}+6.853$$

**Fig 10:**
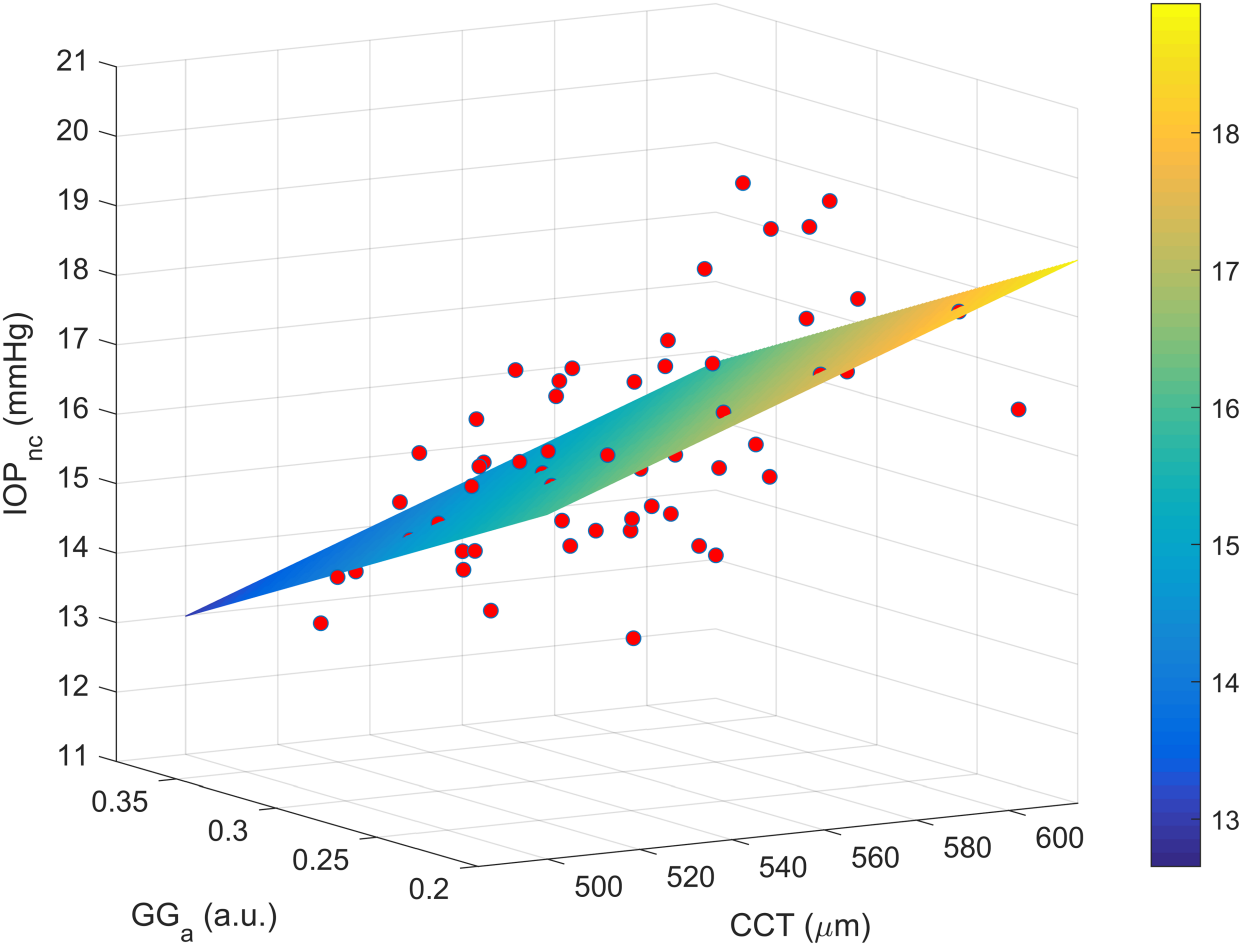
Three-dimensional representation of the equation (3) for all non-corrected IOP measurements.

Fig 10 shows the three-dimensional representation of the equation (3) for all non-corrected IOP values. The forward stepwise regression for corrected IOP was not performed since the corrected values are CCT dependent.

## Discussion

We analysed the influence of the structural parameters on IOP measurements performed with the noncontact tonometer, Corvis ST. Although Goldman applanation tonometry has been used as the gold standard in clinical care, studies have shown that there are no statistically significant differences between readings from a Goldman tonometer and those from Corvis ST [33,34]. Corneal parameters were divided into two groups, macro and micro-structural parameters. Macro-structural analysis included the parameters that are associated with the ocular anatomy whereas micro-structural parameters were related to the internal organisation of the corneal tissue and its physiological changes during lifetime.

The influence of the macro-structural parameters has been extensively explored [7,8,31]. In general, CCT has been reported to be more important for tonometry calibration than CR. In our study, we observed a significant relationship between CCT and IOP (R^2^ = 0.25, *p*<0.001) which is in agreement with the majority of the studies in the literature. Bañeros-Rojas *et al*. [34] obtained an R^2^ of 0.183 for 178 eyes whereas Bao *et al*. [31] obtained an R^2^ of 0.221 for 99 eyes. In both those studies, it was observed that IOP measurements are affected by CCT independently of the noncontact tonometer used. IOP as a function of CR was presented by Bao *et al*. [31], Kohlhaas et al. [8] and Shimmyo *et al*. [35]. Similarly to the results obtained in our study, none of those studies reported a significant relationship between CR and IOP.

In this study, other macro-structural parameters such as AL, ACD and WTW corneal diameter were considered. No statistical significant relationship between them and the measured IOP (p>0.05) was observed. This indicates that CCT is the only macro-structural biometric parameter that plays a key role in ocular tonometry.

Although a large number of studies focused on macro-structural parameters, it is difficult to identify the most precise IOP correction in terms of CCT. Hence, the macro-structure only partially explains the variance of IOP whereas other properties related to the micro-structure also contribute. However, our knowledge about the biomechanical properties of the human cornea *in vivo* is still limited due the lack of means to quantitatively measure it *in-vivo*.

Recently, age has been proposed as a correction factor in applanation tonometry [9,36]. In fact, Corvis ST already allows correcting the IOP with the aid pachymetry-based correction tables including the Dresden correction table [8] and the new Spoerl’s correction table [7], which takes patient age into account. Age certainly affects the corneal tissue [16], however it is not a linear process because it depends on a number of factors such as, environment, ocular anatomy and medical history. Keratoconus and diabetes are two examples of diseases that affect millions of people worldwide and play a significant role on corneal structure and biomechanics [37–39]. Our results showed a statistically significant correlation between IOP and age for the uncorrected IOP with respect to CCT. However, the correlation dropped its significance after IOP correction. Such correlation before IOP correction may be associated to the relationship between CCT and age since it is well known that CCT is age dependent [40,41]. Therefore, for this study, we may conclude that age may not be a good parameter for quantify changes in corneal micro-structure.

In this study, speckle statistics were proposed to describe the organisation of the corneal micro-structure and hence, to analyse its influence on IOP measurements. The analysis of tissue properties by speckle statistics has already been explored in different modalities such as ultrasound [42] and OCT imaging [22,23,43]. Grzywacz *et al*. [22] proposed an automatic statistical procedure based on OCT speckle for a retina with diabetic retinopathy whereas Amini *et al*. [23] recently suggested to use speckle statistics to model the retinal layers.

The GG distribution is well suited to model corneal OCT speckle. A statistically significant relationship between the scale parameter of the GG distribution and the non-corrected IOP (R^2^ = 0.19, *p*<0.001) was observed. The correlation between these two parameters remained significant (R^2^ = 0.14, *p* = 0.002) after CCT influence from the IOP values was removed. A statistically significant correlation was also observed for the ratio of shape parameters before (R^2^ = 0.17, *p*<0.001) and after correcting the IOP (R^2^ = 0.07, *p* = 0.026).

It has been proposed [44,45] that the scale parameter of the GG probability density functions indicates the averaged backscattered power whereas the ratio of the shape parameters is related to the scatter density. We observed that higher IOP values are, in general, associated with a lower GG scale and a higher ratio (*v/p*) of GG shape parameters. This may indicate that an elevated scatter density is related to a stiffer cornea and consequently may contribute to an overestimation of the IOP. The GG probability density function in Fig 9 shows that elevated IOP measurements are associated with a narrower distribution around a centre on a lower intensity.

The information in the micro-structure comes mainly from the collagen fibrils embedded in an extracellular matrix rich in proteoglycans, glycoproteins and keratocytes. Patel *et al*. [46] analysed normal human keratocyte density and observed it varies around 15% between healthy subjects. In addition, they reported that the number of keratocytes in the full-thickness central stroma does not correlate with the central corneal thickness or central stromal thickness. These results suggest there is a substantial variation of the corneal micro-structure in healthy subjects which is not significantly reflected in the macro-structure. However, in their study, it is also mentioned that there is a significant correlation between stromal thickness and keratocyte density. Such observation, it is in agreement with our results since a significant correlation was observed between CCT and the ratio of the shape parameters. However, OCT speckle statistics provide more information about the corneal micro-structure since the scale parameter is independent of the CCT. In addition to the inter-subject variability, corneal micro-structure suffers age-related changes including an increase in the cross-sectional areas of both collagen fibrils and fibrillar molecules which may be due an increase in non-enzymatic cross-linking [16]. There is also a decrease in the inter-fibrillar spacing, related to changes in the proteoglycan composition of the inter-fibrillar matrix. All these micro-structural changes play an important role in corneal biomechanics contributing to a variation of the tissue stiffening among subjects and with age. Our results show that variations on corneal microarchitecture which may have origin in the keratocyte density or in the arrangement of the collagen fibers are reflected in the backscattered light. OCT speckle is sensitive to these changes and its modeling using Generalized Gamma distribution can be used to infer the micro-structural organization of the corneal tissue.

## Conclusion

In this prospective study, we explored the influence of the corneal structure on IOP measurements. The presented experimental findings show that noncontact tonometry is influenced by both macro and micro corneal structure. Central corneal thickness was the only macro-structural parameter that presented a significant influence on IOP measurements. Age did not reveal to be a good indicator of the corneal properties affecting the IOP measurement. Contrarily, the parameters of Generalised Gamma distribution were statistically significantly correlated with the IOP measurements suggesting they can be utilised to analyse and quantitatively evaluate the corneal micro-structure *in vivo*. The proposed technique of statistically modelling OCT speckle introduces a new approach to provide complementary information to better understand the influence of alterations of the collagen framework on ocular tonometry. Nevertheless, further studies with glaucoma subjects are necessary in order to validate a mathematical model for correcting the IOP measurement for factors related to the corneal micro-structure.

## Supporting information

S1 Table Data acquired with IOLMaster, Corvis ST and OCT. Data acquired with IOLMaster (AL, CCT, ACD, CR, WTW), Corvis ST (IOP_c_ and IOP_nc_), OCT (GG_v_, GG_a_, GG_p_) and age.
(XLSX)
